# Cognitive Effects of ThinkRx Cognitive Rehabilitation Training for Eleven Soldiers with Brain Injury: A Retrospective Chart Review

**DOI:** 10.3389/fpsyg.2017.00825

**Published:** 2017-05-22

**Authors:** Christina Ledbetter, Amy Lawson Moore, Tanya Mitchell

**Affiliations:** ^1^Louisiana State University Health Sciences Center, ShreveportLA, United States; ^2^Gibson Institute of Cognitive Research, Colorado SpringsCO, United States; ^3^Research and Development, LearningRx, Colorado SpringsCO, United States

**Keywords:** cognitive training, cognitive rehabilitation, traumatic brain injury (TBI), neuroplasticity, Memory, processing speed, IQ

## Abstract

Cognitive rehabilitation training is a promising technique for remediating the cognitive deficits associated with brain injury. Extant research is dominated by computer-based interventions with varied results. Results from clinician-delivered cognitive rehabilitation are notably lacking in the literature. The current study examined the cognitive outcomes following ThinkRx, a clinician-delivered cognitive rehabilitation training program for soldiers recovering from traumatic brain injury and acquired brain injury. In a retrospective chart review, we examined cognitive outcomes of 11 cases who had completed an average of 80 h of ThinkRx cognitive rehabilitation training delivered by clinicians and supplemented with digital training exercises. Outcome measures included scores from six cognitive skill batteries on the Woodcock Johnson – III Tests of Cognitive Abilities. Participants achieved gains in all cognitive skills tested and achieved statistically significant changes in long-term memory, processing speed, auditory processing, and fluid reasoning with very large effect sizes. Clinically significant changes in multiple cognitive skills were also noted across cases. Results of the study suggest that ThinkRx clinician-delivered cognitive training supplemented with digital exercises may be a viable method for targeting the cognitive deficits associated with brain injury.

## Introduction

Between 2000 and 2016, the US Department of Defense reported nearly 360,000 cases of traumatic brain injury (TBI) among military members (Defense and Veteran’s Brain Injury Center [DVBIC], 2017). Further, TBI is the cause of 1.7 million visits to the emergency room each year resulting in an estimated 60 billion dollars in medical costs and associated lost productivity (Faul et al., 2010). TBIs can range from mild concussions to severe amnesia causing a majority of patients to suffer functional deficits in thinking, sensation, language, and emotion regardless of the cause of the injury (Defense Centers of Excellence for Psychological Health and Traumatic Brain Injury [DCOE] and Defense and Veterans’ Brain Injury Center, 2009). TBI frequently results in impaired executive processing skills which impacts processing speed, reasoning, problem-solving, increased distractibility, and even language impairment (Kinnunen et al., 2011). Memory is the most frequently impaired cognitive skill in patients with brain injury, and evidence suggests that attention skills are at the root of such memory decline (Sohlberg et al., 2000; Cicerone et al., 2005; Gordon et al., 2006). In addition, deficits in verbal delayed memory and set-shifting are also implicated in reduced cognitive functioning after TBI (Karr et al., 2014). In the cases of mild TBI, these deficits may be subtle and difficult to detect with standard neuropsychological testing, but may still interfere with activities of daily living, driving, and work or school performance. Much research on TBI is primarily targeted to civilians, but the cognitive impairments experienced as a result of TBI do not appear to differ among combat and non-combat related TBI survivors, nor between blast-related and other etiologies of brain trauma (Belanger et al., 2009).

Although methods for treating the emotional and social impact of brain injury vary, approaches to treating the cognitive deficits associated with TBI have included attention and memory training using mnemonic strategies (Thickpenny-Davis and Barker-Collo, 2007) and external cuing supports (Wilson et al., 2005). Following an extensive review of the literature by two working groups, attention process training and working memory training for the remediation of cognitive impairments due to TBI emerged as clinical recommendations from the Defense Centers of Excellence for Psychological Health and Traumatic Brain Injury [DCOE] and Defense and Veterans’ Brain Injury Center (2009), as well as from the Cognitive Rehabilitation Task Force of the American Congress of Rehabilitation Medicine (Haskins et al., 2012). The literature is just beginning to proliferate, but previous research on cognitive training for soldiers with TBI is encouraging. For example, empirical support for specific attention and memory training procedures has been documented for letter cancelation tasks with distractions (Sohlberg et al., 2000; Tiersky et al., 2005; Sinotte and Coelho, 2007), simultaneous completion of two cognitive tasks (Cicerone et al., 2005; Serino et al., 2007), and retrieval practice with verbal paired associates (Sumowski et al., 2010).

With the alarming incidence of brain injury among military members, there is a critical need for a systematically applied set of therapeutic services that restore executive functioning skills. The most effective interventions will be those that target the root causes of cognitive dysfunction and utilize strategies based on the current knowledge of brain plasticity following injury. The efficacy of one such training program, called ThinkRx (Gibson, 2003), was previously explored in several studies with children and teens with learning disabilities. In the first study (Gibson et al., 2015), the experimental group (*n* = 31) achieved greater gains than the control group (*n* = 30) in associative memory, working memory, processing speed, auditory processing, visual processing and fluid reasoning as measured by the Woodcock Johnson – III with medium to large effect sizes. In the second study (Carpenter et al., 2016), significant differences were found between the treatment group (*n* = 20) and the control group (*n* = 19) on seven cognitive outcome variables including IQ score. Initial findings from magnetic resonance imaging (MRI) research on the ThinkRx cognitive training program revealed greater global efficiency for the treated group versus the control group, as well as statistically significant correlations between changes in functional connectivity and changes in cognitive test scores (Ledbetter et al., 2016). Observational data on the ThinkRx program also revealed significant improvements across multiple cognitive skills for clients with TBI (*n* = 273), including mean standard point gains of 18 points on tests of long-term memory and 12 points on tests of working memory (Wainer and Moore, 2016).

In addition to support for clinician-delivered interventions, the feasibility of computer-based cognitive skills training for soldiers with brain injury is gaining support as well (Lebowitz et al., 2012; Bogdanova et al., 2016). However, a gap in the cognitive training research on TBI and ABI is the use of a combined approach to cognitive training program delivery. In one study, supplementing the clinician-delivered ThinkRx cognitive training program with computer-based exercises was compared to delivering the program solely by a clinician-delivered method in a randomized controlled trial with children (Moore et al., 2016). Results indicated similar results between both delivery methods on all variables expect for long-term memory. Due to the need to provide a scalable and affordable cognitive rehabilitation training intervention, these findings were encouraging. The current study builds on these prior findings by examining the benefits of combined delivery methods of the ThinkRx program in remediating the cognitive deficits associated with TBI and acquired brain injury (ABI). The mechanism of change in cognitive rehabilitation training is grounded in neuroplasticity research and the evidence of experience-induced cortical plasticity (see Huang, 2009). That is, cortical functioning changes in response to experience (Buonomano and Merzenich, 1998; Schwartz and Begley, 2003). The evidence of functional map expansion following ThinkRx training found in Ledbetter et al. (2016) supports this theory of training-induced plasticity for the current study.

The purpose of the current study was to examine the outcomes of a sample of soldiers with brain injury who had participated in a pilot program using ThinkRx, the clinician-delivered cognitive training intervention, supplemented with computer-based cognitive training tasks. The research questions for the current study were (1) Is there a statistically significant difference in performance measures of cognitive skills following ThinkRx cognitive training? (2) Are the differences in cognitive skill performance clinically significant? and (3) Do participants report real-life benefits from cognitive training? Based on prior research, we hypothesized that there would be statistically significant differences between pretest and post-test scores, that at least three cognitive skill changes would be clinically significant for each participant, and that participants would report real-life benefits following cognitive training.

## Materials and Methods

### Participants

Review of cognitive rehabilitation training records from concurrent referrals to an occupational therapy clinic from a warrior transition unit (WTU) at a large Army base in the Western part of the United States revealed 15 cases of transitioning active-duty soldiers diagnosed with brain injury who had volunteered for a pilot cognitive rehabilitation training program using ThinkRx. All soldiers were assigned to the WTU based on comprehensive eligibility determination that each needed greater than 6 months of complex medical care and rehabilitation due to moderate or severe injuries prior to transitioning back to active duty service or to civilian life outside the military (Department of the Army, 2009). Thus, all brain injuries in this sample were classified as moderate-to-severe. Four participants who volunteered and initiated training did not complete the intervention. Three were discharged from the military and one returned to full military duty. The remaining participants included a purposive sample of soldiers (*n* = 11) who had sustained a brain injury within the prior 3 years while serving in the military, and who were at least 3 months post injury. Time since injury ranged from 3 to 38 months (*M* = 11.2, *SD* = 10.5). Two injuries were penetrating, eight injuries were diffuse axonal injuries, and one injury was acquired from an aneurysm. Participants ranged in age from 25 to 46 (*M* = 33.7, *SD* = 7.7). All of the participants were male. Thirty-six percent of participants were identified as Caucasian (*n* = 4), 27% were identified as Black (*n* = 3), 27% were identified as Asian (*n* = 2), and 9% were identified as Native American (*n* = 1). Demographic and clinical characteristics of the participants are presented in **Table 1**.

**Table 1:** Participant demographics.

Case	Gender	Age	Type of Injury	Time since injury (months)
A	M	35	Blunt	7
B	M	27	Penetrating	19
C	M	42	Blast	5
D	M	29	Blunt	10
E	M	25	Blunt	3
F	M	28	Blast/Penetrating	10
G	M	26	Blast	5
H	M	42	Fall	38
I	M	36	Blast	10
J	M	33	Blast	5
K	M	47	Aneurysm	24

### Procedures

The retrospective chart review was approved by the Institutional Review Board (IRB) at the Gibson Institute of Cognitive Research in accordance with exempt research Category 4 of 45 CFR 46.101(b)(4). Cognitive rehabilitation training records were reviewed from concurrent participation in the ThinkRx pilot program at an occupational therapy clinic between January 2010 and August 2010. The original unpublished pilot study had been approved by the ethics review committee at LearningRx. All participants had provided written informed consent in accordance with the Declaration of Helsinki. For the current study, relevant information from participant files was compiled in a database of demographic and outcome variables. Because the pretest and post-test assessments were objective measures, the quality of the outcome data and the subsequent reliability of the test results can be considered high (Worster and Haines, 2004). The use of retrospective chart reviews is supported across disciplines and clinical research areas due to the benefits of generating hypotheses for future prospective studies through analysis of rich existing data (Gearing et al., 2006).

### Intervention

The intervention used in the current study was adapted from a clinic-based cognitive training program, ThinkRx (Gibson, 2003). The one-on-one cognitive training program is delivered by clinicians who use 23 game-like mental tasks with multiple variations that increase in difficulty as training progresses. The program utilizes varying levels of intensity and loading of multiple tasks to remediate attention, auditory processing, executive processing, logic and reasoning, long-term memory, working memory, processing speed, and visual processing skills. Trainers add a metronome and deliberate distractions to the sessions to keep intensity high and the focus demanding. Participant and clinician workbooks include a detailed progression through the levels of each procedure to ensure continuity in treatment implementation and mastery of the tasks.

ThinkRx is traditionally delivered four or 5 days per week in a training center. However, accommodating the hefty time commitment and scheduling demands for soldiers recovering from brain injury can be challenging. Thus, the current study utilized digital cognitive training tasks to supplement the trainer-delivered intervention. Participants attended three 60-min rehabilitation training sessions in clinic each week for 24 weeks and were instructed to complete an additional hour per week of supplemental computer-based training in their barracks using Brainskills. Brainskills software delivers training tasks similar to those facilitated by the clinician in the one-on-one environment. Assigned as homework, the Brainskills tasks can be completed from a personal computer at times most convenient to the participants. Brainskills is web-based, enabling the participants to access and use it from any computer with a graphics card and speakers. Participants were scheduled to receive 72 h of one-on-one cognitive training and 24 h of computer-based training, for a total of 96 h. The intervention was delivered by master’s level clinicians including a speech and language pathologist and a licensed cognitive rehabilitation therapist.

**FIGURE 1:**
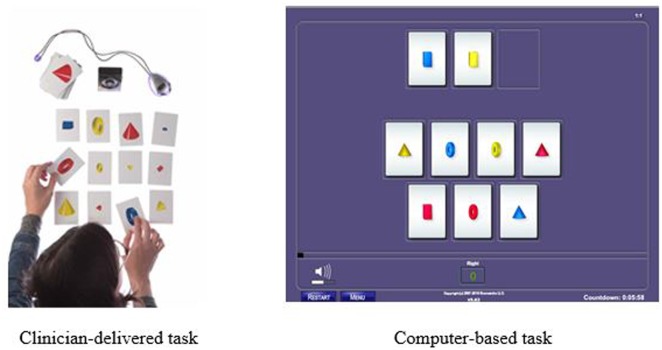
**Example of Reasoning BrainCards fluid reasoning training task: Clinician-delivered task and computer-based task**.

An example of one of the 23 training tasks is called *Reasoning BrainCards*, a clinician-delivered cognitive training procedure that requires participants to identify a three-card group from a set of nine or 12 cards. Each card has four features: shape, color, orientation, and size. For one level of the task, the participant must identify three cards that share a common variable. For another level, the participant is only shown two cards and must identify which card is needed to complete the three-card group. In the digital version of the same task, the computer automatically generates the card groups. This task develops logic and reasoning, comprehension, working memory, processing speed, attention, and visual processing. The version of the clinician-delivered task and the corresponding computer-based task is illustrated in **Figure 1**.

**FIGURE 2:**
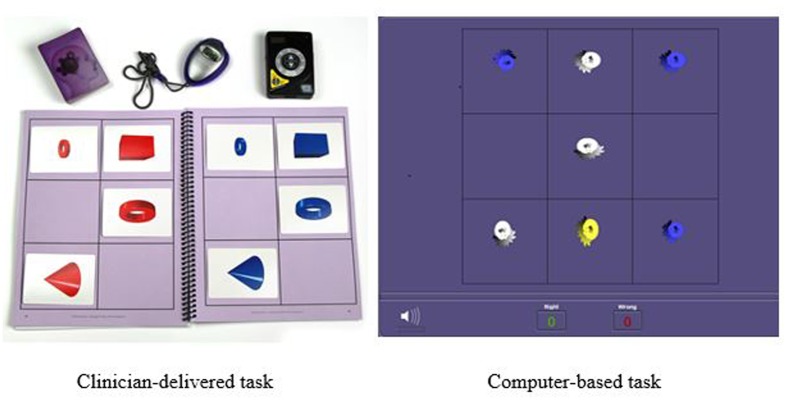
**Example of Memory Match memory training task: Clinician-delivered task and computer-based task**.

Another training procedure is called *Memory Match* (shown in **Figure 2**) which targets working memory, processing speed, visual discrimination, and sustained attention. In the clinician-delivered version of the task, the trainer randomly arranges cards containing cones, rings, or boxes into a pattern that the participant may study for 3 s. After, the trainer covers his workboard and the participant must reproduce the same pattern on his own workboard while simultaneously counting aloud to the beat of a metronome. There are nine progressively more difficult levels for this procedure with 34 total variations. In the digital version of this task, the participant studies the pattern presented on the screen and then must select the correct pattern from a set of possible responses and distractors. There are 29 difficulty levels and 87 variations in the digital version.

### Cognitive Assessment Measures

All participants were administered selected measures from the Woodcock Johnson – III –Tests of Cognitive Abilities (Woodcock et al., 2001) before and after the cognitive training intervention. Based on the Cattell–Horn–Carroll theory of cognitive abilities, the Woodcock Johnson – III (WJ III) is designed to measure eight broad categories of cognitive functioning along with 69 specific abilities. The WJ III has been normed on adults in the same age range (*n* = 1843) as the sample for the current study. Test–retest reliability coefficients range from 0.70 to 0.94 for this age group, and internal consistency reliability estimates fall in the 0.80s and 0.90s. For the current study, six batteries were selected for pre and post-testing. These batteries were consistent with the skills typically identified as compromised in participants with TBI. The tests were administered to participants by a master’s-level clinician trained in the use of the Woodcock Johnson – III. Results were scored and analyzed by a doctoral level psychologist. A description of the tests and the associated cognitive skills is found in **Table 2**.

**Table 2:** Outcome variables and Woodcock Johnson – III tests.

Variable	WJ III test	Description
Long-term memory	Visual auditory learning	Participant learns a rebus; then recalls and recites the association between the pictures and words.
Visual processing	Spatial relations	Participant visually matches individual puzzle pieces to a completed shape.
Auditory processing	Sound awareness	Participant performs rhyming, deletion, substitution, and reversal of sounds to create/identify new words.
Fluid reasoning	Concept formation	Participant applies inductive rules to a set of shapes and indicates the rule that differentiates the sets.
Processing speed	Visual matching	In 3 min, participant identifies and circles pairs of matching numbers in each row.
Working memory	Numbers Reversed	Participant hears a list of numbers and repeats them in reverse order.
General Intellectual Ability (GIA)	Composite score for *g*	Weighted composite of seven WJ III tests.

The assessment tasks on the Woodcock Johnson – III are qualitatively different than the intervention tasks. The tests are designed to measure skills in isolation, but the training tasks target multiple overlapping skills. For example, memory was assessed using two WJ III tests. First, the Visual-Auditory Learning test measured delayed recall and semantic memory. After learning a set of pictures that each represents a word, the participant must recall the associations by reading the picture sentences aloud. Next, short term and working memory were assessed using the Numbers Reversed test. Participants were asked to repeat a set of numbers in reverse order from which they were presented. These tasks differed from the training memory training tasks. During the intervention, memory was trained through a variety of complex visual and auditory tasks such as Memory Match (shown in **Figure 2**) and Memory Digits—a training procedure requiring participants to study a playing card with a nine-space grid of numbers and blanks while simultaneously counting aloud to a metronome beat, adding a given number to the numbers on the card, and then reciting the new numbers in order without a visual prompt. The Memory Digits procedure not only targets working memory but also attention, visualization, and visual span.

### Data Collection and Analysis

#### Test Data

Test data were abstracted from individual patient files by a master’s-level research associate familiar with chart reviews. Although test reports were included in the charts, the abstractor retrieved the raw test data and input them into Compuscore software to obtain standard scores, *W* scores, and percentiles for each participant on the Woodcock Johnson – III tests. A second research associate reviewed the raw data and the converted standardized scores. This process is consistent with recommended procedures for retrospective chart reviews to ensure accuracy of the test data (Worster and Haines, 2004). The data were then analyzed by a doctoral-level psychologist using IBM SPSS 23 software to conduct both descriptive and inferential analysis. Paired samples *t*-tests were conducted on pairs of standard scores from the Woodcock Johnson – III tests to determine statistical significance of gains from pretest to post-test. Then, individual test data were analyzed for clinically significant change and the Reliable Change Index (RCI). We used a two-part procedure to analyze the clinical significance of the changes for each participant. The first gauge of clinically significant change is movement from a pretest score in a clinical range to a post-test score in a range one would expect from healthy individual. Following the Jacobson–Truax Method (Jacobson and Truax, 1991), we first calculated the healthy population cut point for each measure; that is, the value above which a score is most likely to fall in a distribution of scores for people without TBI. Then, we calculated a RCI for each participant to determine if the magnitude of the pretest to post-test change in *W* scores was statistically reliable.

#### Qualitative Data

Qualitative data were abstracted from individual patient files by trained research associates who were blind to the study’s hypothesis to reduce subjectivity (Worster and Haines, 2004). Data were acquired from clinician notes on intake and exit interviews, and on training session records. Data were typed onto an EXCEL template, recording each comment as a direct quote notated with the corresponding participant’s first and last initials. The spreadsheet was transferred to the investigators for thematic analysis.

## Results

### Training Compliance

Eleven participants completed the intervention. Although the participants were scheduled to complete 60–90 total hours of training (a minimum of 60 training hours is required to complete the ThinkRx program), the actual training hours completed by each participant ranged from 54 to 101 over the course of the study due to variability in treatment compliance and attendance. According to notes in the charts, five participants found it difficult to complete the supplemental computer-based training because of headaches, vision problems, pre-existing seizures, or technical problems with a personal computer. **Table 3** illustrates the training compliance/completion for participants who were included in the review. Although Case I remained in the program for the duration and returned for post-testing, he did not complete the minimum number of 60 training hours required for the ThinkRx program. Thus, his results were excluded from group significance testing and the collective descriptions of clinical change, but still included in the individual analyses for clinically significant change.

**Table 3:** Completion of training hours.

Case	Clinician-delivered training hours	Supplemental digital training hours	# of total training hours completed
A	71	5	76
B	72	29	101
C	72	25	97
D	72	8	80
E	70	3	73
F	62	5	67
G	72	5	77
H	45	15	60
I	51	3	54^∗^
J	71	16	87
K	54	20	74

*^∗^Did not complete minimum program requirement of 60 h.*

### Assessment Results

#### Statistical Significance

Participants achieved gains in all skills measured. Using the traditional alpha of *p* < 0.05, the gains were statistically significant with very large effect sizes for all variables except visual processing. After Bonferroni correction for multiple comparisons and adjusting the alpha to *p* < 0.007, results remained statistically significant for general intellectual ability, *t*(9) = 7.8, *p* < 0.001, and for four of the six individual cognitive skills: long-term memory, *t*(9) = 4.2, *p* = 0.002; auditory processing, *t*(9) = 3.5, *p* = 0.007; fluid reasoning, *t*(9) = 6.9, *p* < 0.001; and processing speed, *t*(9) = 4.1, *p* = 0.003. Positive and nearly significant gains were made in working memory, *t*(9) = 3.1, *p* = 0.012 and visual processing, *t*(9) = 2.1, *p* = 0.067. **Table 4** illustrates the mean pretest and post-test standardized *W* scores, standard deviations, and the effect sizes (Cohen’s *d*) indicating the magnitude of change from pretest to post-test. Effect sizes over 0.9 are considered “very large” and indicate the practical significance of the score gains (Cohen, 1969). The table also illustrates the pre and post training mean percentiles for each skill tested, revealing large improvements across skills. The largest improvements were seen in GIA, long-term memory, working memory, and auditory processing, followed by fluid reasoning and processing speed. The smallest improvement was seen in visual processing.

**Table 4:** Significance testing of change in cognitive skills.

WJ III test	Pre *W* score Mean (*SD)*	Post *W* score Mean (*SD)*	Difference	*p*	Effect size Cohen’s *d*	Pre Percentile Mean (*SD)*	Post Percentile Mean (*SD)*
General Intellectual Ability (GIA)	513.7 (9.4)	527.6 (8.5)	13.9^∗^	0.000	2.5	32 (17)	63 (19)
Long-term memory (COG 2)	498.7 (7.9)	510.0 (10.1)	11.3^∗^	0.002	1.3	35 (20)	64 (19)
Visual processing (COG 3)	506.4 (10.9)	513.5 (9.8)	7.1	0.067	0.66	45 (26)	60 (23)
Auditory processing (ACH 21)	505.8 (13.2)	519.6 (13.1)	13.8^∗^	0.007	1.1	30 (26)	59 (29)
Fluid reasoning (COG 5)	518.3 (14.5)	532.7 (13.4)	14.4^∗^	0.000	2.2	55 (26)	76 (17)
Processing speed (COG 6)	506.5 (13.9)	518.3 (8.8)	11.8^∗^	0.003	1.3	19 (18)	37 (19)
Working memory (COG 7)	518.3 (24.2)	542.2 (20.4)	23.9	0.012	0.99	39 (30)	68 (25)

*^∗^significant at Bonferroni-corrected alpha *p* < 0.007.*

Due to the variation in treatment hours completed by each participant, we wanted to determine if the variation related to training gains. This information was critical to determine future study protocols. Because the sample size was too small for a regression analysis, we ran a one-sample *t*-test on residuals calculated by covarying out the total number of training hours. After Bonferroni correction for multiple comparisons and adjusting the alpha to *p* < 0.007, results of the analyses indicated significant differences between participants on long-term memory, *t*(9) = 6.6, *p* < 0.001; visual processing, *t*(9) = 9.3, *p* < 0.001; and auditory processing, *t*(9) = -3.8, *p* = 0.004. Differences based on the covariate ‘number of training hours’ were not significant for working memory, *t*(9) = -3.3, *p* = 0.01; fluid reasoning, *t*(9) = 2.7, *p* = 0.03; processing speed, *t*(9) = -0.30, *p* = 0.77; or IQ score, *t*(9) = 3.2, *p* = 0.01.

#### Clinical Significance

**FIGURE 3:**
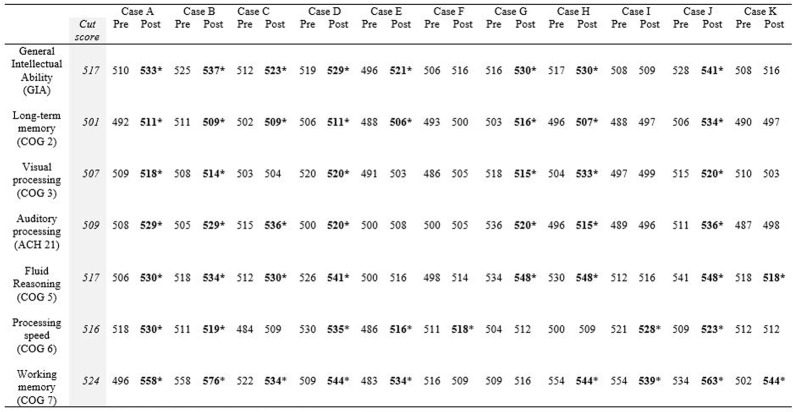
**Cut score thresholds and clinically significant change**.^∗^Post-test scores meeting threshold for clinically significant change are in bold.

**Figure 3** illustrates the clinical significance of the changes from pretest to post-test on each cognitive assessment. As a reference, the cut scores for each skill are included. The post-test scores meeting the cut score threshold are annotated with an asterisk. As noted in the figure, 9 of the 11 working memory post-test scores, eight of the General Intellectual Ability composite post-test scores, the long-term memory post-test scores, and the fluid reasoning post-test scores met the threshold for 95% probability of occurring in a normal population.

**Table 5** shows the RCI of each measure by participant. The descriptors listed underneath each RCI are determined by the following: participants are described as “recovered” if their post-test score meets the cut score threshold for healthy populations *and* the RCI was statistically reliable (RCI > 1.96). RCIs greater than 1.96 but final scores that do not meet the cut score threshold are described as “improved.” RCIs between 1.96 and -1.96 are described as “unchanged” and RCIs less than -1.96 are described as “deteriorated.” Ten of the eleven participants obtained a significant clinical change and significant RCI on General Intellectual Ability (GIA)—the composite measure of cognitive skills tested—indicating overall recovery or improvement effects from the intervention. The 11th participant (Case I) did not complete the minimum number of training hours, so the remaining narrative excludes his results. With the exception of the GIA composite scores, 31 of the 60 possible subtest score changes across participants were clinically significant and revealed recovery or clinical improvement (52%). Twenty-four scores remained clinically unchanged (40%), and five deteriorated from pretest levels (8%). Clinical improvement and recovery rates on individual subtest scores ranged from 20 to 90%. On fluid reasoning, 90% (9 of 10) showed clinical improvement or recovery. On auditory processing, 80% (8 of 10) showed clinical improvement or recovery. On working memory, 50% (5 of 10) showed clinical improvement or recovery. On processing speed, 40% (4 of 10) showed clinical improvement or recovery. The lowest improvement or recovery percentages were on long-term memory with 30% (3 of 10) and visual processing with 20% (2 of 10).

**Table 5:** Reliable change index by case and variable.

Variable	Case A	Case B	Case C	Case D	Case E	Case F	Case G	Case H	Case I	Case J	Case K
General Intellectual Ability	**13.5^∗^ R**	**6.6^∗^ R**	**7.1^∗^ R**	**5.5^∗^ R**	**13.7^∗^ R**	**5.5^∗^ I**	** 7.6^∗^ R**	** 8.4^∗^ R**	0.59 U	** 7.6^∗^ R**	** 5.2^∗^ I**
Long-Term Memory	**2.5^∗^ R**	-0.23 D	0.82 U	0.59 U	**2.1^∗^ R**	0.82 U	0.59 U	1.3 U	1.2 U	** 3.7^∗^ R**	0.82 U
Visual Processing	1.3 U	0.88 U	0.18 U	0.0 U	1.8 U	**2.8^∗^ I**	-0.43 U	**5.3^∗^ R**	0.29 U	0.73 U	-1.3 D
Auditory Processing	**7.9^∗^ R**	** 7.3^∗^ R**	** 8.4^∗^ R**	** 6.1^∗^ R**	** 2.4^∗^ I**	1.5 U	-4.9 D	** 7.6^∗^ R**	** 2.6^∗^ I**	** 9.5^∗^ R**	** 4.4^∗^ I**
Fluid Reasoning	**5.5^∗^ R**	**3.3^∗^ R**	**4.1^∗^ R**	**3.1^∗^ R**	**3.3^∗^ I**	**3.3^∗^ I**	**2.9^∗^ R**	** 3.7^∗^ R**	0.91 U	**1.6^∗^ R**	0.0 U
Processing Speed	**1.9^∗^ R**	1.2 U	**4.3^∗^ I**	0.77 U	**4.6^∗^ R**	1.1 U	1.2 U	1.5 U	1.1 U	**2.2^∗^ R**	0.0 U
Working Memory	**5.5^∗^ R**	1.4 U	1.1 U	**2.7^∗^ R**	**3.9^∗^ R**	-0.62 D	0.54 U	-0.93 D	-1.3 D	**2.5^∗^ R**	**3.9^∗^ R**

*^∗^Significant RCI > 1.96. R, recovered; I, improved; U, unchanged; D, deteriorated. Recovered and improved indicated in bold.*

### Qualitative Self-Report Results

The chart review revealed qualitative data were documented by clinicians for 6 of the 11 participants. Therefore, the sample for the qualitative analysis was limited to those six participants. Thematic analysis of self-reported benefits after training revealed three primary themes: increased confidence and perseverance, improved attention, and improved memory. Increased confidence and perseverance were reported by 100% of participants (*n* = 6) and included examples such as “I’m sticking with something even when it gets too hard,” “I had enough confidence to take the GRE for graduate school,” “I’m able to make plans and stick with them,” “This training helped me make the decision to go back to school,” and “I’m now facing my weaknesses face to face.” Improvements in attention were reported by 67% of participants (*n* = 4) and included examples such as “I’m not in a haze. I am able to organize and stay focused,” “My attention span is longer,” “I’m able to stay on task longer,” and “I’m able to read for longer periods of time and comprehend what I’m reading.” Improvements in memory were also reported by 67% of participants (*n* = 4) and included examples such as “It’s easier to remember all the steps needed to finish a project,” “I feel ahead of the curve in remembering items like my wallet and phone before I leave the house,” “My memory is better. I’m able to remember a list for the store without writing it down,” and “I need reminders on my phone much less.”

In addition to the primary themes identified, other examples of self-reported benefits included improved social skills and tolerance levels, renewed interest in learning, better math and language skills, and better organization. One participant referred to the program as “a bright light in a dark space” and another said it was “the most helpful thing I have experienced in my life.”

## Discussion

This retrospective chart review examined the cognitive outcomes for 11 soldiers with brain injury who had completed the ThinkRx cognitive rehabilitation training pilot program at a military warrior transition unit in the Western United States. The participants achieved improvements in all cognitive skills tested. The gains made by participants were statistically significant for general intellectual ability and for four of the six individual cognitive skills: fluid reasoning, auditory processing, long-term memory, and processing speed. Although not statistically significant after Bonferroni correction, participants did achieve positive gains in working memory and visual processing as well. The changes in percentiles certainly reflect those gains. The large and very large effect sizes are noteworthy considering the challenge in finding an effect with small samples.

Further, the clinical significance of the findings is encouraging. In alignment with the important trend of reporting clinically significant change for psychological outcome research (Atkins et al., 2005), we examined a quantifiable measure of participants’ improvement or recovery. Overall, eight of the 10 participants included in the analysis could be classified as “recovered” and additional two participants could be classified as “improved” given their RCI scores. That is, all ten of the participants who completed the minimum required training hours saw clinically significant improvement overall. Nine participants saw clinically significant changes in three to six areas of cognitive ability.

The qualitative findings align with the cognitive testing outcomes. Self-reports revealed that participants were cognizant and appreciative of functional improvements in memory, attention span, confidence and grit, organizational skills, math and language skills, and even social skills by the end of the program. The improvements in daily functioning are encouraging for the use of cognitive training in brain injury rehabilitation efforts and suggest that the benefits of cognitive training may extend beyond the improvement of cognitive test scores.

These results suggest that the ThinkRx cognitive training program may be a viable intervention for targeting the cognitive skill deficits associated with brain injury. However, the usefulness of supplementing one-on-one cognitive training with computer-based training remains unclear. Indeed, the variability in computer-based training hours completed at home by the participants complicated the interpretation of the results. Because there were statistically significant differences in results based on the number of computer-based training hours completed, it is still unclear whether the use of supplemental computer-based training is feasible. Four of the five participants who completed less than 15 h of supplemental computer-based training identified the reasons as headache and vision problems associated with using the program. Whether these effects resulted from visual stress (Wilkins, 2005) or pre-existing photosensitivity from the TBI is unknown. The fifth participant suffered from seizures prior to beginning the program and was instructed by his physician to stop using the computer altogether. Although headache and fatigue experienced by the participants are consistent with prior research on computer-based cognitive training for TBI (Lebowitz et al., 2012), they are also commonly reported symptoms of TBI sufferers in general (Formisano et al., 2009). Because the remaining participants in the current study enjoyed the computer-based aspect of the program without symptoms of headache or fatigue, a prospective study with a larger sample size is indicated before conclusions can be drawn.

There were some limitations of the current study, including the lack of randomization or a control group. However, a retrospective chart review is exploratory in its design and does not imply causation. Indeed, it is interesting to note the magnitude of the effect sizes in gains, suggesting strong support for evaluating the use of the program in a controlled study with soldiers recovering from TBI. Another limitation of the current study is the variation in training hours completed by the participants. This variation may have reduced treatment fidelity, making it challenging to determine the usefulness of the computer-based supplemental training. A third limitation of the study is the small sample size which reduces the statistical power of the analyses. However, the use of clinically significant change indices lends robustness to the findings and the study outcomes support conducting a prospective study with a larger sample size. It should also be noted that due to the protected nature of military medical records, the researchers were only given access to the cognitive training and assessment records from the original pilot study and not to the entire medical chart. This limits the ability to consider detailed medical history—including details about each brain injury—in assessing the outcomes. Finally, it is important to note that some readers may be concerned over the heterogeneous etiology of the brain injuries in the sample. However, prior research suggests that the cognitive sequelae do not differ significantly between blast-related and other mechanisms of moderate-to-severe brain injury (Belanger et al., 2009). We contend that the improvements noted across participants suggest that the cognitive training gains were not dependent upon etiology. That is, gains and improvements were found across etiologies. This finding supports a future prospective study with homogenous groups of participants with penetrating injuries, diffuse axonal injuries, and focal injuries. Although the results are encouraging and certainly consistent with results seen in clinical use of the ThinkRx program with clients recovering from TBI (Wainer and Moore, 2016), it is premature to speculate that all people with brain injury would respond favorably to the intervention. The intense nature of the training may preclude those with the severest of brain injuries from engaging in the training tasks to the extent needed to effect cortical change. The participants in the current study were motivated to complete the cognitive training program with the goal of returning to active duty military service. Participants with lesser motivation may not see similar gains.

A strength of the current study was that the results were taken from a clinical population in a military transition unit which lends external validity to the intervention. The use of standardized testing measures lends objectivity to the results and enables future comparison to other intervention outcomes. The inclusion of qualitative outcomes adds an element of ecological validity to the study by suggesting that participants recognized practical improvements from the training intervention. Future studies should include a control group, a measure of fatigue with an evaluation of its impact on training progress, and health history of the participants that may influence treatment outcomes.

## Conclusion

The purpose of the current retrospective study was to evaluate the outcomes of cognitive rehabilitation training using supplemental computer-based cognitive training with an established clinician-delivered cognitive training program (ThinkRx) with soldiers recovering from brain injury. Although the small sample size and inconsistency in the number of training hours completed precludes a decision about the feasibility of the hybrid approach to cognitive training, all participants realized improvements in cognitive skills which suggests that the program was beneficial. Indeed, prospective studies are warranted to determine the optimal combination of cognitive training delivery methods for participants with brain injury.

## Author Contributions

All three authors made substantial contributions to the conception and design of the article and to the acquisition, analysis, and interpretation of the data. All authors worked on the drafts and revisions to add important intellectual content. Specifically, AM conducted the literature review, performed the data analyses, and authored the results section. CL reviewed the data analyses, crafted the discussion section, and conducted the final editing. TM performed the data collection and co-authored the methods section. All authors approved the submitted version and agree to be accountable for all aspects of the work to ensure its accuracy and integrity.

## Conflict of Interest Statement

CL is a member of the scientific advisory board for the research institute associated with the intervention but does not accept payment beyond travel reimbursement. AM operates the research institute associated with the intervention used in the current study but does not have a financial interest in the outcomes of the research. TM is related the creator of the intervention evaluated in the current study and has a 10% financial interest in the company. The reviewer ARD and the handling Editor declared their shared affiliation, and the handling Editor states that the process nevertheless met the standards of a fair and objective review.

## References

[B1] Atkins D. C. Bedics J. D. McGlinchey J. B. Beauchaine T. P. (2005). Assessing clinical significance: does it matter which method we use? *J. Consult. Clin. Psychol.* 73 982–989. 10.1037/0022-006X.73.5.982 16287398

[B2] Belanger H. G. Kretzmer T. Yoash-Gantz R. Pickett T. Tupler L. A. (2009). Cognitive sequelae of blast-related versus other mechanisms of brain trauma. *J. Int. Neuropsychol. Soc.* 15 1–8. 10.1017/S1355617708090036 19128523

[B3] Bogdanova Y. Yee M. K. Ho V. T. Cicerone K. D. (2016). Computerized cognitive rehabilitation of attention and executive function in acquired brain injury: a systematic review. *J. Head Trauma Rehabil.* 31 419–433. 10.1097/htr.0000000000000203 26709580 PMC5401713

[B4] Buonomano D. V. Merzenich M. M. (1998). Cortical plasticity: from synapses to maps. *Annu. Rev. Neurosci.* 21 149–186. 10.1146/annurev.neuro.21.1.149 9530495

[B5] Carpenter D. M. Ledbetter C. Moore A. L. (2016). LearningRx cognitive training effects in children ages 8-14: a randomized controlled study. *Appl. Cogn. Psychol.* 30 815–826. 10.1002/acp.3257 27867257 PMC5108426

[B6] Cicerone K. D. Dahlberg C. Malec J. F. Langenbahn D. M. Felicetti T. Kneipp S. (2005). Evidence-based cognitive rehabilitation: Updated review of the literature from 1998 through 2002. *Arch. Phys. Med. Rehabil.* 86 1681–1692. 10.1016/j.apmr.2005.03.024 16084827

[B7] Cohen J. (1969). *Statistical Power Analysis for the Behavioral Sciences.* San Diego, CA: Academic Press.

[B8] Defense Centers of Excellence for Psychological Health and Traumatic Brain Injury [DCOE] and Defense and Veterans’ Brain Injury Center (2009). *Cognitive Rehabilitation for Mild Traumatic Brain Injury: Summary of Clinical Recommendations.* Available at: https://www.dcoe.mil/files/Cognitive_Rehabilitation_Therapy_for_Traumatic_Brain_Injury_RTC.pdf

[B9] Defense and Veteran’s Brain Injury Center [DVBIC] (2017). *DoD Worldwide Numbers for Traumatic Brain Injury.* Available at: http://dvbic.dcoe.mil/files/tbi-numbers/DoD-TBI-Worldwide-Totals_2016_Feb-17-2017_v1.0_2017-04-06.pdf

[B10] Department of the Army (2009). *Warrior Transition Unit Consolidated Guidance.* Available at: http://wct.army.mil/documents/policies.html#WCTP_Manuals

[B11] Faul M. Xu L. Wald M. M. Coronado V. G. (2010). *Traumatic Brain Injury in the United States: Emergency Department Visits, Hospitalizations, and Deaths.* Atlanta, GA: Centers for Disease Control and Prevention.

[B12] Formisano R. Bivona U. Catani S. D’Ippolito M. Buzzi M. G. (2009). Post-traumatic headache: facts and doubts. *J. Head Pain* 10 145–152. 10.1007/s10194-009-0108-4 PMC3451986 19294482

[B13] Gearing R. E. Mian I. A. Barber J. Ickowicz A. (2006). A methodology for conducting retrospective chart review research in child and adolescent psychiatry. *J. Can. Child Adolesc. Psychiatry* 15 126–134. PMC2277255 18392182

[B14] Gibson K. (2003). *ThinkRx: Cognitive Training Procedures Workbook.* Colorado Springs, CO: LearningRx.

[B15] Gibson K. Carpenter D. Moore A. L. Mitchell T. (2015). Training the brain to learn: beyond vision therapy. *Vis. Dev. Rehabil.* 1 119–128.

[B16] Gordon W. A. Zafonte R. Cicerone K. Cantor J. Brown M. Lombard L. (2006). Traumatic brain injury rehabilitation: State of the science. *Am. J. Phys. Med. Rehabil.* 85 343–382. 10.1097/01.phm.0000202106.01654.61 16554685

[B17] Haskins E. C. Cicerone K. Dams-O’Connor K. Eberle R. Langenbahn D. Shapiro-Rosenbaum A. (2012). *Cognitive Rehabilitation Manual: Translating Evidence-Based Recommendations Into Practice.* Reston, VA: American Congress of Rehabilitation Medicine.

[B18] Huang J. C. (2009). Neuroplasticity as a proposed mechanism for the efficacy of optometric vision therapy and rehabilitation. *J. Behav. Optom.* 20 95–99.

[B19] Jacobson N. S. Truax P. (1991). Clinical significance: a statistical approach to defining meaningful change in psychotherapy research. *J. Consult. Clin. Psychol.* 59 12–19. 10.1037/0022-006X.59.1.12 2002127

[B20] Karr J. E. Areshenkoff C. N. Duggan E. C. Garcia-Barrera M. (2014). Blast-related mild traumatic brain injury: a bayesian random-effects meta-analysis on the cognitive outcomes of concussion among military personnel. *Neuropsychol. Rev.* 24 428–444. 10.1007/s11065-014-9271-8 25253505

[B21] Kinnunen K. M. Greenwood R. Powell J. H. Leech R. Hawkins P. C. Bonnelle V. (2011). White matter damage and cognitive impairment after traumatic brain injury. *Brain* 134 449–463. 10.1093/brain/awq347 21193486 PMC3030764

[B22] Lebowitz M. S. Dams-O’Connor K. Cantor J. B. (2012). Feasibility of computerized brain plasticity-based cognitive training after traumatic brain injury. *J. Rehabil. Res. Dev.* 49 1547–1556. 10.1682/JRRD/2011.07.0133 23516058

[B23] Ledbetter C. Faison M. O. Patterson J. (2016). “Correlation of Cognitive Training Gains and Resting State Functional Connectivity,” *Presented at the Society for Neuroscience*, San Diego, CA.

[B24] Moore A. L. Ledbetter C. Carpenter D. M. (2016). “Intensive, metronome-based, 1-on-1 cognitive training improves cognitive skills in children,” in *Poster Presented at Society for Neuroscience 2016*, San Diego, CA.

[B25] Schwartz J. Begley S. (2003). *The Mind and the Brain: Neuroplasticity and the Power of Mental Force.* New York, NY: Harper Collins.

[B26] Serino A. Ciaramelli E. Santantonio A. Malag S. Servadei F. Làdavas E. (2007). A pilot study for rehabilitation of central executive deficits after traumatic brain injury. *Brain Injury* 21 11–19. 10.1080/02699050601151811 17364515

[B27] Sinotte M. P. Coelho C. A. (2007). Attention training for reading impairment in mild aphasia: a follow-up study. *NeuroRehabilitation* 22 303–310. 17971621

[B28] Sohlberg M. M. McLaughlin K. Pavese A. Heidrich A. Posner M. (2000). Evaluation of attention process training and brain injury education in persons with acquired brain injury. *J. Clin. Exp. Neuropsychol.* 22 656–676. 10.1076/1380-3395(200010)22:5;1-9;FT656 11094401

[B29] Sumowski J. F. Wood H. G. Chiaravalloti N. Wylie G. R. Lengenfelder J. Deluca J. (2010). Retrieval practice: A simple strategy for improving memory after traumatic brain injury. *J. Int. Neuropsychol. Soc.* 16 1147–1150. 10.1017/S1355617710001128 20946709

[B30] Thickpenny-Davis K. L. Barker-Collo S. L. (2007). Evaluation of a structure group format memory rehabilitation program for adults following brain injury. *J. Head Trauma Rehabil.* 22 303–313. 10.1097/01.HTR.0000290975.09496.93 17878772

[B31] Tiersky L. A. Anselmi V. Johnston M. V. Kurtyka J. Roosen E. Schwartz T. (2005). A trial of neuropsychologic rehabilitation in mild-spectrum traumatic brain injury. *Arch. Phys. Med. Rehabil.* 86 1565–1574. 10.1016/j.apmr.2005.03.013 16084809

[B32] Wainer H. Moore A. (2016). *LearningRx Client Outcomes and Research Results.* Available at: http://download.learningrx.com/results-report.pdf

[B33] Wilkins A. J. (2005). Visual stress in neurological disease. *Adv. Clin. Neurosci. Rehabil.* 4 22–23.

[B34] Wilson B. A. Emslie H. Quirk K. Evans J. Watson P. (2005). A randomized control trial to evaluate a paging system for people with traumatic brain injury. *Brain Inj.* 19 891–894. 10.1080/02699050400002363 16296571

[B35] Woodcock R. W. McGrew K. S. Mather N. (2001). *Woodcock Johnson - III.* Itasca, IL: Riverside Publishing.

[B36] Worster A. Haines T. (2004). Advanced statistics: understanding medical record review (MRR) studies. *Acad. Emerg. Med.* 11 187–192. 10.1197/j.aem.2003.03.002 14759964

